# Energetic and Biomechanical Contributions for Longitudinal Performance in Master Swimmers

**DOI:** 10.3390/jfmk5020037

**Published:** 2020-06-05

**Authors:** Daniel A. Marinho, Maria I. Ferreira, Tiago M. Barbosa, José Vilaça-Alves, Mário J. Costa, Ricardo Ferraz, Henrique P. Neiva

**Affiliations:** 1 Department of Sport Sciences, University of Beira Interior, 6201-001 Covilhã, Portugal; mines_ferreira@sapo.pt (M.I.F.); ricardompferraz@gmail.com (R.F.); henriquepn@gmail.com (H.P.N.); 2 Research Centre in Sports, Health and Human Development, CIDESD, 6201-001 Covilhã, Portugal; barbosa@ipb.pt (T.M.B.); josevilaca@utad.pt (J.V.-A.); mario.costa@ipg.pt (M.J.C.); 3 Department of Sport Sciences, Polytechnic Institute of Bragança, 5300-253 Bragança, Portugal; 4 Department of Sport Sciences, Exercise and Health Sciences, University of Trás-os-Montes and Alto Douro, 5001-801 Vila Real, Portugal; 5 Department of Sport Sciences, Polytechnic Institute of Guarda, 6300-559 Guarda, Portugal

**Keywords:** training, swimming, efficiency, lactate, oxygen uptake

## Abstract

Background: The current study aimed to verify the changes in performance, physiological and biomechanical variables throughout a season in master swimmers. Methods: Twenty-three master swimmers (34.9 ± 7.4 years) were assessed three times during a season (December: M_1_, March: M_2_, June: M_3_), in indoor 25 m swimming pools. An incremental 5 × 200 m test was used to evaluate the speed at 4 mmol·L^−1^ of blood lactate concentration (sLT), maximal oxygen uptake (*VO_2max_*), peak blood lactate ([La-]peak) after the test, stroke frequency (*SF*), stroke length (*SL*), stroke index (*SI*) and propelling efficiency (*η_p_*). The performance was assessed in the 200 m front crawl during competition. Results: Swimming performance improved between M_1_, M_2_ (2%, *p* = 0.03), and M_3_ (4%, *p* < 0.001). Both sLT and *VO_2max_* increased throughout the season (4% and 18%, *p* < 0.001, respectively) but not [La-]peak. While *SF* decreased 5%, *SL*, *SI* and *η_p_* increased 5%, 7%, and 6% (*p* < 0.001) from M_1_ to M_3_. Conclusions: Master swimmers improved significantly in their 200 m front crawl performance over a season, with decreased *SF,* and increased *SL, η_p_* and *SI.* Despite the improvement in energetic variables, the change in performance seemed to be more dependent on technical than energetic factors.

## 1. Introduction

The participation of athletes older than 35 years in training and competition has been increasing over the last years, particularly in swimming [1]. Master swimmers strive to maintain or even improve upon the performance achieved at younger ages, seeking to counter the normal decline associated with ageing [2]. Initially, involvement in exercise and sport is mainly due to social reasons, such as enjoyment, travel, and stress relief, in addition to health benefits and the improvement of physical fitness [3,4]. However, performance maximization arises as a goal, whereby the identification of the factors that might predict performance with high accuracy is important in this age group. In this sense, training control should be a priority by using regular evaluation tests to provide relevant information for coaches and swimmers. For instance, incremental exercise testing is a procedure used to determine submaximal and maximal physiological variables, such as maximal oxygen uptake (*VO_2max_*) and lactate threshold (LT), and biomechanical variables, such as stroke frequency (*SF*), stroke length (*SL*), index stroke (*SI*) and propelling efficiency of the arm stroke (*η_p_*). These variables are usually used in research to provide reliable and valid data to monitor the effects of training in elite swimming [5,6]. However, only a few studies have focused on the assessment of training and performance in master swimmers [7]. Master swimmers are a fascinating model of exceptionally successful ageing, and therefore are highly deserving of scientific attention, complementing the scarcity of knowledge and data about them. 

The literature has that, in young and elite swimmers, performance is strongly linked to energetic variables, as these are dependent on biomechanical profile and motor strategies adopted by the swimmers [5]. Among the energetic factors that are important for swimming performance are the highest blood lactate concentration in post-exercise condition ([La-]peak), the velocity at 4 mmol·L^−1^ of blood lactate concentration (sLT), and maximal oxygen uptake (*VO_2max_*) [8,9,10]. Following and during exercise in adulthood, in general both lactate production and removal are reduced when compared to younger counterparts [11,12]. Ageing causes changes in body composition that alters the muscle structure and reduces the ability to perform exercises requiring strength and power [13]. Moreover, a progressive reduction in *VO_2max_* appears to be the primary mechanism associated with declines in endurance performance with age, followed by a reduction in LT (i.e., the exercise intensity at which blood lactate concentration increases significantly) [2]. *VO_2max_* declines approximately 10% per decade after age 25–30 years in healthy sedentary adults of both genders [14]. Beyond this, muscle strength and power also inexorably decline with ageing [13]. Therefore, it seems that there is a decline in maximal aerobic and anaerobic power and capacities with increasing age [15,16]. Moreover, it was suggested that the decrease in performance with age was greater in long-term events than short-term events, which could mean a higher rate of decline of aerobic-related variables than anaerobic-related variables [17]. Thus, swimming training might play a fundamental role in preventing this declining trend and allow the maintenance or improvement of sports performance, in addition to the individual’s metabolic functions.

The goal of a competitive swimmer is to travel a given distance as fast as possible [18], whereby mean swimming speed and time are the best measures for swimming performance [19]. It is known that swimming speed and. therefore, time performed is dependent on the relationship between the *SL* and the *SF,* and *SI* and *η_p_* variables [5,18]. These variables are strongly related to technical parameters of swimming [5]. For example, at a given speed, greater *SI* and *SL* indicates a swimmer with a more efficient swimming technique [20]. Moreover, an increase in *η_p_* value represents increased efficiency of the work that is effectively used to propel the swimmer forward and depends on the anthropometric characteristics of the swimmer and his/her technical skills [21]. Contrarily to other sports activities where minimal differences in efficiency are observed among subjects with different technical abilities, the efficiency of swimming is deeply influenced by training. Thus, it becomes important to understand the possible changes in the technical parameters of swimming training. It was reported that *SI* and *η_p_* increase with training in elite swimmers [6]. However, the analysis of how kinematic parameters change throughout a season is scarce regarding master swimmers.

Scientific literature on master swimmers simply reports cross-sectional data about their physiological and biomechanical characteristics [22]. Longitudinal data are reduced when compared to their young and elite counterparts, focusing exclusively on performance [17] and energy cost [14] adaptations based on race time’s progression. To the best of our knowledge, it seems there is a lack of scientific evidence regarding master swimmers’ energetic and biomechanical adaptations throughout a training season. Therefore, the current research aimed to assess the performance, physiological and biomechanical parameters in master swimmers in three distinct periods over one season. It was hypothesized that an improvement in performance, energetic and biomechanical variables would occur throughout the season.

## 2. Materials and Methods

### 2.1. Subjects

Twenty-three master swimmers (34.9 ± 7.4 years) participated in the study, twelve males (aged 35.0 ± 7.5-years) and eleven females (aged 34.7 ± 7.3 years). Table 1 presents the main anthropometrics and performance characteristics. The conversion of times into FINA points was made using the procedure suggested by Daly & Vanlandewijck [23]. Male and female swimmers, aged 30–50 years, were recruited by detailed announcements at a local swimming club. The following inclusion criteria were considered: (i) male or female; (ii) 25–50 years-old (iii) have a background as swimmer participating in national swimming events; (iv) be engaged in a systematic master swimming program. The exclusion criteria included: (i) any physical challenge; (ii) musculoskeletal injury, pathology or condition; (iii) pregnancy; (iv) more than three consecutive weeks of absence during the follow-up period. All subjects gave their written informed consent before participation. The study was approved by University of Beira Interior ethics committee (under the project d975, December 2015) and is in accordance with the Declaration of Helsinki. 

### 2.2. Study Design

**Figure 1:**
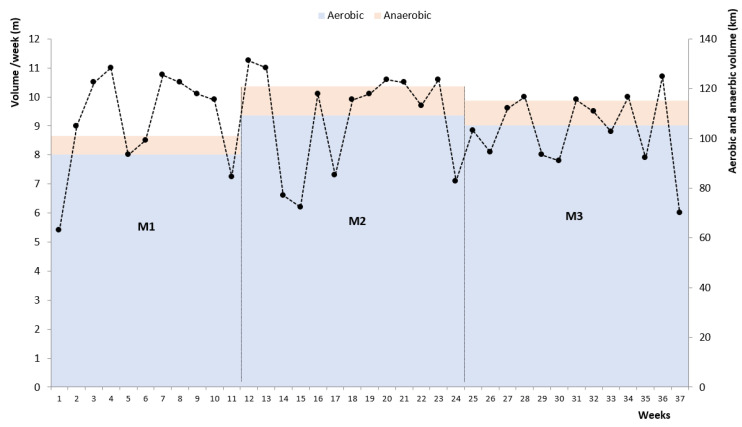
Training volume per week throughout the season (dashed line and dots), and distribution of aerobic and anaerobic training volume in each period of evaluation (M_1_, M_2_ and M_3_).

A longitudinal research design was carried out, so that swimmers were evaluated in three different time periods over a season: December (M_1_), March (M_2_) and June (M_3_). The evaluations were performed in the 11th, 24th, and 37th weeks of training, respectively. Swimming training consisted of three sessions per week, involving low, medium, and high aerobic tasks, sprint work and technical drills. Weekly training averaged 9.0 ± 1.7 km wk^−1^. Throughout the season, the training of swimmers presented intensity corresponding to aerobic (M_1_: 92.81%; M_2_: 90.35%; M_3_: 91.36%) and anaerobic capacity (M_1_: 7.19%; M_2_: 9.65%; M_3_: 8.64%) (Figure 1). The training process was always accompanied by the research team, with the coach of the team. The distinction between aerobic and anaerobic loads was carried out taking into account the considerations of Maglischo [24] and using the same procedure as previous studies [6,8]. In each instance, the 200 m front crawl performance, sLT, [La-]peak, *VO_2max_*, *v_200_*, *SF*, *SL*, *SI*, *η_p_* were collected.

### 2.3. Performance Data Collection

Swimming performance was assessed during official short course competitions in local, regional and national competitions. The 200 m front crawl times were obtained from the official competition results (https://www.swimrankings.net), that took place in the week before the step test evaluation (M_1_, M_2_, and M_3_).

### 2.4. Energetic and Biomechanical Data Collection

An incremental 5 × 200 m step test, in a 25 m pool, was used to evaluate the swimmers’ energetic adaptation [25]. Push-off starts were used in each task. The starting speed was set at approximately 80% of the swimmers’ personal best time at the point of evaluation, representing a low training pace [25]. The best performance at each evaluation instance was assessed based on official competition results, performed in the week before evaluation (https://www.swimrankings.net). The velocities increased 5% in each step, ensuring that the final task was performed at maximal speed. The rest period was set at 2 min maximum, to allow the assessment of physiological variables and ensure that the swim intensity incrementally increased from the first to the last repetition of the swimming task. Underwater pacemaker lights (GBK-Pacer, GBK Electronics, Aveiro, Portugal), located on the bottom of the pool, were used to control the swimming speed and help swimmers keep an even pace along each lap during the first 4 repetitions of 200 m. The last was performed as quickly as possible. Elapsed time for each trial was measured with a stopwatch (SEIKO S141) by an exporter evaluator, as a backup. 

Oxygen uptake (*VO_2_*) was measured with a backward extrapolation technique immediately after each trial (Kb4^2^, Cosmed, Rome, Italy). Swimmers were instructed to breathe during the last cycle before touching the wall. After finishing the trial, the swimmer leaned on the wall, while an operator fixed a portable mask on his face during the recovery period. No breathing cycle was made until the portable mask was on the swimmer’s face. The *VO_2_* value (in mL·kg^−1^·min^−1^), reached during each step of the protocol, was estimated using the backward extrapolation of the O_2_ recovery curve. *VO_2max_* was considered to be the mean value in the 6 s after the *VO_2_* detection during the recovery period [26]. The first measurement of *VO_2_* values, before the highest *VO_2_* measurement, was not considered, because it corresponded to the device adaptation to the sudden change of respiratory cycles and O_2_ uptake. The device adaptation never exceeded 2 s [6,26]. Fingertip capillary blood samples were collected before the step test and after the last 200 m front crawl repetition, at the 1st, 3rd, 5th, and 7th minutes of recovery. Samples were then analyzed for blood lactate concentrations (Accusport, Boherinnger Mannheim, Germany). [La-]peak was considered to be the highest blood lactate concentration in the post-exercise condition [10]. The individual sLT was obtained by interpolation of the average lactate value (4 mmol·L^−1^) on the exponential curve of lactate/speed relationship [27,28].

Swimming speed (s) is the ratio of the distance to the elapsed time needed to travel that distance, and it was measured considering the mean value obtained in each lap (measured between 5 m and 20 m):

(1) $$s=\frac{d}{t}$$

where s is the swimming speed (in m·s^−1^), *d* is the distance (in m) and *t* (in s) is the time required to travel that distance. 

The biomechanical profile was determined based on the measurement of *SF* (in Hz), *SL* (in m), *SI* (in m^2^·c^−1^·s^−1^) and *η_p_* (in %). *SF* was recorded manually from three consecutive stroke cycles in the middle of each lap, during each trial, using a chrono-frequency meter (Golfinho Sports MC 815, Aveiro, Portugal). Then, *SF* values were converted to International System Units (i.e., Hz). *SL* was estimated as being [19]:

(2) $$SL=\frac{s}{SF}$$

where *SL* is the stroke length (in m), s is the swimming speed (in m·s^−1^), and *SF* is the stroke frequency (in Hz). *SI* is considered as one of the swimming stroke efficiency indexes and was computed as [20]:

(3) SI=s·SL

where *SI* is the stroke index (in m^2^·c^−1^·s^−1^), s is the swimming speed (in m·s^−1^) and *SL* is the stroke length (in m). *η_p_* was also estimated as being [29]:

(4) $$\eta_{p}=\left(\frac{s\cdot 0.9}{2\pi \cdot SF\cdot l}\right)\cdot \frac{2}{\pi }$$

where *v* is the swimming speed (in m·s^−1^), (multiplied by 0.9 to take into account that, in front crawl, about 10% of forward propulsion is produced by the legs), *SF* is the stroke frequency (in Hz) and *l* is the arm’s length (in m). The *l* is computed trigonometrically measuring the arm’s length and considering the average elbow angles during the in-sweep of the arm pull, as reported by Zamparo [23]. Equation (4) is, properly speaking, the Froude efficiency. The difference between Froude and propelling efficiency is that the first does not take into account the effect of internal mechanical work on total mechanical work production. As reported by Zamparo et al. [29], at the range of swim speed verified in these swimmers, internal mechanical work is rather low and can be neglected. So propelling efficiency becomes very similar to Froude efficiency. 

### 2.5. Statistical Procedures

The normality of all distributions was verified using the Shapiro-Wilks tests. Parametric or non-parametric tests were selected accordingly. Mean plus one standard deviation and quartiles were computed for each time period. The relative frequency of variation (i.e., the percentage of change) between time periods was also reported. Pearson correlation coefficients were determined between performance values, energetic and biomechanical variables. Data variation was assessed with ANOVA repeated measures, followed by the Bonferroni post-hoc test, as well as the Wilcoxon Signed-Rank Test, to assess differences between time periods (M_1_-M_2_; M_1_-M_3_; M_2_-M_3_). The level of statistical significance was always set at *p* ≤ 0.05.

## 3. Results

Improvements were observed throughout the season, with a decrease in 200 m front crawl time from M_1_ to M_3_ (M_1_-M_2_: −1.9%, *p* = 0.03; M_2_-M_3_: −2.2%, *p* = 0.01, and M_1_-M_3_: −4.1%, *p* < 0.001). Table 2 presents the individual performance values in the 200 m front crawl, in each evaluation moment.

Analysing the individual modifications in M_1_-M_2_, the performance improvement in eleven of the twenty-three swimmers was concomitant with an increase in *SL* and a decrease in *SF*, two swimmers increased *SF* and decreased *SL* and five swimmers increased both parameters (*SL* and *SF*). The remaining five swimmers presented an increase of time between M_1_-M_2_. In M_2_-M_3_, the performance improvement in 6 of the 23 swimmers was concomitant with an increase in *SL* and a decrease in *SF*, nine swimmers increased *SF* and decreased *SL* and one swimmer increased both parameters (*SL* and *SF*). The remaining seven swimmers presented a decrease in performance. Between the first and the last time period, the performance improvement in 7 of the 23 swimmers was concomitant with an increase in *SL* and decrease in *SF*, seven swimmers increased *SF* and decreased *SL* and two swimmers increased both parameters (*SL* and *SF*). The remaining seven swimmers presented a decrease in performance between M_1_-M_3_.

**Figure 2:**
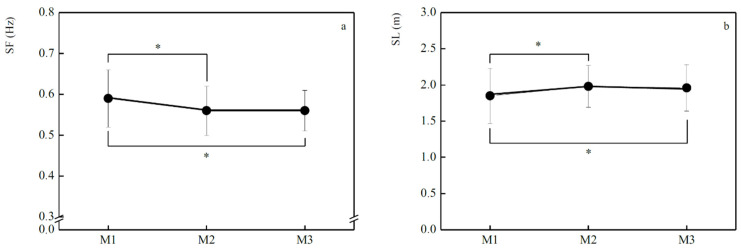
Mean ± SD values of stroke frequency (SF: **a**) and stroke length (SL: **b**) in each evaluation moment (M_1_, M_2_, M_3_). * significant differences in SF between M_1_-M_2_ (*p* < 0.001), M_1_-M_3_ (*p* = 0.04) and in SL between M_1_-M_2_ (*p* = 0.02) and M_1_-M_3_ (*p* = 0.04).

Figure 2 presents the variation in biomechanical variables (*SF* and *SL*). Data reported a decrease in *SF* from M_1_-M_2_ (−5.1%, *p* < 0.001), remained unchanged between M_2_-M_3_ (0.1%) and decreased from M_1_-M_3_ (−5.1%, *p* = 0.04) (Figure 2a). In contrast, *SL* exhibited an increase between M_1_-M_2_ (5.7%, *p* = 0.02) and M_1_-M_3_ (5.1%, *p* = 0.04). From M_2_-M_3_, *SL* presents a non-significant decrease (−0.5%) (Figure 2b).

**Figure 3:**
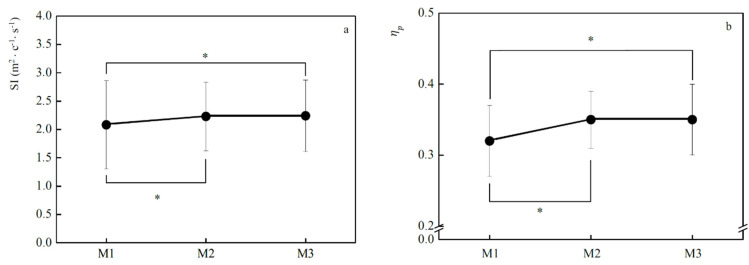
Mean ± SD values of stroke index (SI: **a**) and propelling efficiency (*η_p_*: **b**) in each evaluation moment (M_1_, M_2_, M_3_). * significant differences in SI between M_1_-M_2_ (*p* < 0.001), M_1_-M_3_: (*p* = 0.04) and in *η_p_* between M_1_-M_2_ (*p* < 0.001) and M_1_-M_3_ (*p* < 0.001).

The values of *SI* and *η_p_* are represented in (Figure 3). Concerning *SI* (Figure 3a) significant increases were observed among M_1_-M_2_ (5.4%, *p* < 0.001) and M_1_-M_3_ (6.8%, *p* = 0.04). For M_2_-M_3_, there is no significant increase in *SI* (1.4%). Finally, *η_p_* (Figure 3b) presents significant increases between M_1_-M_2_ (6.4%, *p* < 0.001) and M_1_-M_3_ (6.3%, *p* < 0.001). For M_2_-M_3_, there is no significant decrease in *η_p_* (−0.1%). In all the biomechanics variables, no differences were found between M_2_-M_3_.

**Figure 4:**
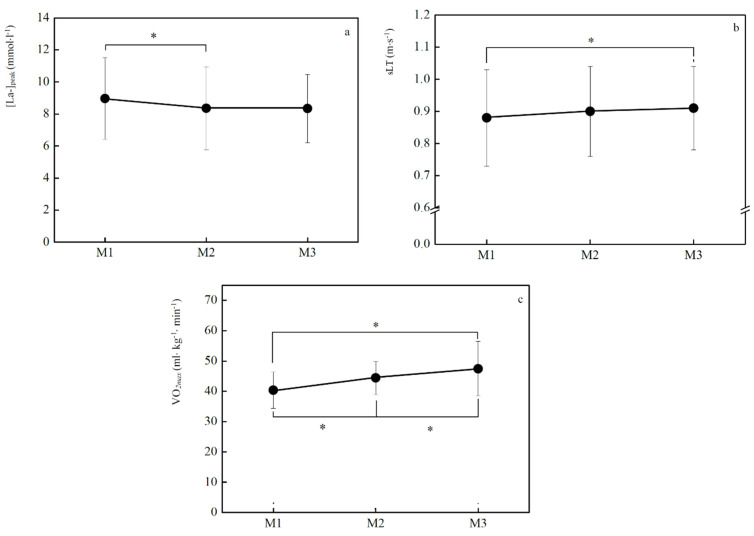
Mean ± SD values of peak lactate concentration ([La-]peak: **a**), speed at 4 mmol L^−1^ (sLT: **b**) and maximal oxygen uptake (*VO_2max_*: **c**) in each evaluation moment (M_1_, M_2_, M_3_). * significant differences in [La-]peak between M_1_-M_2_ (*p* = 0.04); in sLT between M_1_-M_3_ (*p* < 0.001); and in *VO_2max_* between M_1_-M_2_ (*p* < 0.001), M_2_-M_3_ (*p* = 0.03) and M_1_-M_3_ (*p* < 0.001).

Figure 4 presents energetic adaptations throughout the season. [La-]peak decreased significantly from M_1_-M_2_ (−8.8%, *p* = 0.04) and no differences were found between the other evaluation moments (Figure 4a). The sLT increased from M_1_-M_3_ (3.5%, *p* < 0.001), but remained unchanged from M_1_-M_2_ (1.8%) and M_2_-M_3_ (1.7%) (Figure 4b). The *VO_2max_* increased from the first to the last M (M_1_-M_2_: 10.0%, *p* < 0.001; M_2_-M_3_: 7.3%, *p* = 0.03; M_1_-M_3_: 18.0%, *p* < 0.001) (Figure 4c).

The energetic variable with a higher percentage of change throughout the season was *VO_2max_* (18.0% between M_1_-M_3_), while SI was the bio-mechanic variable with a higher percentage of change (6.8% between M_1_-M_3_). There were no significant correlations between the changes from M_1_-M_2_, M_2_-M_3,_ and M_1_-M_3_ in the 200 m front crawl performance time and the *SF*, *SL*, *SI*, *η_p_*, [La-]peak, sLT, and *VO_2max_*. Nevertheless, a positive and significant correlation was found between changes in *SF* and 200 m time between M_1_-M_3_ (*r* = 0.49, *p* = 0.02).

## 4. Discussion

The purpose of this study was to analyse the changes in performance, energetic and biomechanical profiles of master swimmers throughout a season. The main results were the significant changes observed in performance throughout the season, and in energetic (except [La-]peak) and biomechanical profiles in master swimmers.

### 4.1. Performance

There was a performance improvement between evaluation moments due to biomechanical and energetic changes throughout the season. Considering that the major changes were recorded from M_1_-M_3_, it seemed that the 200 m performance improved significantly mainly due to the improvement of *SI* and *VO_2max_*. There was an improvement in swimming technique, corroborated by the significant increase of *SL, SI, η_p_*, and the decrease in *SF.* The *SL*, *SI* and *η_p_*, are recognized as good propulsive efficiency indicators, and can be used to evaluate progress in technique level. It was expected that, in swimmers with a lower performance level, *SL* and the effectiveness of propulsive force represented important factors affecting performance [30], especially in the first months of the training season. During these months, the aerobic loads allow swimming at low velocities, focusing on technical aspects of the stroke mechanics and, thus, improving technical ability. In fact, the aerobic training focus can be supported by the increase in sLT from M_1_-M_3_, an important variable to monitor the aerobic capacity of the swimmers [6,9]. Moreover, a large increase was found in *VO_2max_* throughout the season and this could be relevant to explain the performance improvement, together with biomechanical variables. 

### 4.2. Biomechanics

In the biomechanical variables, significant changes were observed throughout the season, especially for M_1_-M_2_ and M_1_-M_3._ No differences were found in any of the biomechanical variables between M_2_-M_3_. This fact may be ascribed to the concept of detraining before M_1_. In the transition between two seasons (off-season), if the athletes do not practice, they probably lose performance. In this case, the tests in M_1_ were performed when the subjects had few training sessions, after the off-season. So it seems evident that at the beginning of the season the changes should be more accentuated, especially regarding the specific aspects of swimming technique, such as *SF* and *SL.* Moreover, the individual response to a training regimen seems to depend to a great extent on one’s initial performance level and the possibility for a performance improvement is higher as the initial level is lower. 

The significant decrease in *SF* between M_1_-M_2_ and M_1_-M_3_ might mean that, with training, swimmers have learned to perform a more effective stroke and do not need to do so many strokes, highlighted by the changes in the other biomechanical variables. *SL* exhibits a significant increase between M_1_-M_2_ and M_1_-M_3._ The increase of *SL* is generally related to a more forceful and effective stroke [31], revealing an improvement the swimming technique. Swimmers comprising the sample are very heterogeneous in relation to their swimming experience: we have ex-swimmers with participations in national championships when they were young and individuals who started swimming a few years ago. These “recent swimmers” will necessarily have less technical skill than ex-swimmers so, with training, these subjects may be more able to present a larger improvement on swimming technique. The significant enhancement found in *SI* may be explained by the increase in the swimming speed and *SL.* Finally, the significant increase found in *η_p_* may be due to the relationship between *η_p_* and *SF*: lower values of *SF*, for a given speed, lead to higher values in *η_p_* [31] and increased propelling efficiency. Moreover, the significant correlations between the changes in the 200 m front crawl time and the changes in *SF*, seems to suggest that a better performance appears to be dependent on a lower *SF*. These results highlight the role of both *SL* and *SF* for overall performance, even in master swimmers. 

Favaro et al. [32] obtained higher values of *SF* (0.65 ± 0.17 Hz) and *SI* (2.32 ± 0.57 m^2^·c^−1^·s^−1^) compared to ours. The type and intensity of effort may explain these differences. Thus, the race accomplished in Favaro’s study was 50 m distance, at maximal intensity, so, since it is a shorter distance, a higher swimming speed is expected at the expense of the increase of *SF* and not of *SL*. In another study, the subjects swam 50 m (in a 50 m long swimming pool) at constant *v* and *SF* and repeated the swim at three to four different speeds, self-selected by them [31]. Once the distance was shorter the speed achieved was higher (1.29 ± 0.19 m·s^−1^) as was the *SF* (0.65 ± 0.17 Hz) compared to the current study. Moreover, Zamparo [31] used the average time taken to complete five strokes to calculate *SF*, while in this study we used the average time taken to complete three strokes. Lower *SF* values (0.41 ± 0.06 Hz) were found by Zamparo et al. [22], perhaps due to the different intensity used to perform the test (0.93 ± 0.10 m·s^−1^). Thus, at submaximal intensity, speed is achieved by a smaller *SF* and a larger *SL* (2.27 ± 0.25 m) [22]. This highlights the biomechanical differences that are caused by the different level of the master swimmers [16,30] and the anthropometric characteristics of the swimmers, namely the arm length [22,31]. Furthermore, the different tests used in the literature can influence the biomechanical variables. For instance, shorter testing distances could result in higher swimming speeds, attained with higher SF or/and SL.

### 4.3. Energetics

No significant increase in [La-]peak was found throughout the season, despite the increase in performance. The consistency of the [La-]peak values is related to a similar anaerobic contribution throughout the season. This could be related to the increase in swimming efficiency and with the increase in sLT and *VO_2max_*, that could show an increased aerobic fitness. In this way, the better swimming performances found in M_2_ and M_3_ could be resultant from a greater aerobic and similar anaerobic contributions. To the best of our knowledge, no other study tried to understand energetic variables changes throughout a season in master swimmers and so we were not able to compare the data assessed during the step test with previous research, specifically the [La-]peak values. 

The sLT is important for determining the aerobic capacity of the swimmers and it was demonstrated in elite swimmers that it can be improved with training [6,9]. In the present study, sLT increases from the beginning to the end of the season. Generally, in young and elite swimmers, most gains in sLT occur in the early months of the beginning of the season, due to an increase in training volume [33,34]. This is a result of training-induced adaptations which increase the muscle’s ability to produce energy aerobically [34], thus, reducing the rate of muscle glycogen use and lactate production [34]. The results obtained in the master swimmers, that were slightly different from M_1_ to M_3_, could be due to the higher prevalence of aerobic workouts during their training throughout the season (and not only at the beginning of the season), instead of strength, speed, and power training that occurs in young and elite swimmers [35]. The significant increase in *VO_2max_* that resulted in the variable with higher percentage of changes throughout the season corroborates the idea mentioned previously. 

Studies performed with elite swimmers and university swimmers showed that *VO_2max_* remained unchanged throughout a season [6,36]. However, in master swimmers, *VO_2max_* is lower compared with elite swimmers [11], enabling a wide margin of improvement. Maybe because of the decline in physiological systems throughout the lifespan and/or the lower intensity training status, the training season caused the great changes found from M_1_ to M_2_ and from M_2_ to M_3_ in the current study. The increase in *VO_2max_* could be fundamental to performance, augmenting the participation of the aerobic metabolism during maximal efforts and avoiding an excessive production of blood lactate that can lead to the inhibition of contraction of muscle fibers (due to decreased pH), decreasing performance. The increase of aerobic partial contribution was evidenced before [37] and is likely to be related to the high percentage of workout focused on aerobic intensity [37]. 

## 5. Conclusions

Master swimmers significantly improved their 200 m front crawl performance over a season. In the first months of the training season there was an improvement in swimming efficiency, by decreasing *SF,* and increasing *SL, SI* and *η_p_*. The performance improvement throughout the season was partially explained by the changes in SF. It was also evidenced that each swimmer used the most freely chosen combination to reach higher performances throughout the season. Although we found improvement in energetic factors throughout the season, in this age-group performance seems to be more dependent on technical than energetic factors.

## Figures and Tables

**Table 1:** Mean and SD values of anthropometrics and performance characteristics of male swimmers, female swimmers, and all participants.

Participants	Height (m)	Body Mass (kg)	200 m Front Crawl (s)	FINA Points 200 m Front Crawl
Male (*n* = 12)	1.75 ± 0.06	74.81 ± 7.70	170.42 ± 27.77	315.00 ± 128.60
Female (*n* = 11)	1.63 ± 0.05	58.52 ± 5.41	200.72 ± 25.02	254.30 ± 110.80
All (*n* = 23)	1.69 ± 0.06	66.67 ± 6.65	185.20 ± 31.51	286.02 ± 121.71

**Table 2:** Individual performance values in the 200 m front crawl (s) of male (m) and (f) female swimmers, in each evaluation moment (M_1_, M_2_, M_3_).

	200 m Time (s)		
Swimmer	M_1_	M_2_	M_3_
m1	158	153	152
m2	152	162	158
m3	224	229	211
m4	188	181	179
m5	232	242	220
m6	165	162	162
m7	169	167	167
m8	144	141	140
m9	159	162	162
m10	150	148	147
m11	188	186	183
m12	175	172	174
Mean ± SD	175.33 ± 28.26	175.42 ± 30.91	171.21 ± 24.24
f1	192	193	191
f2	183	180	185
f3	231	208	205
f4	210	205	200
f5	218	211	205
f6	207	207	195
f7	207	195	185
f8	220	211	197
f9	248	233	231
f10	171	163	168
f11	170	166	166
Mean ± SD	205.18 ± 24.47	197.45 ± 20.97	193.45 ± 18.12
Total (m + f); Mean ± SD	189.61 ± 30.06	185.96 ± 28.36	181.87 ± 23.91
